# Clinical and virological features of asymptomatic and mild symptomatic patients with SARS‐CoV‐2 Omicron infection at Shanghai Fangcang shelter hospital

**DOI:** 10.1002/iid3.1033

**Published:** 2023-09-27

**Authors:** Lin Zhang, Xiaoyu Kang, Liangliang Wang, Rui Yan, Yanglin Pan, Jiuping Wang, Zhangqian Chen

**Affiliations:** ^1^ Department of Internal Medicine Central Medical Branch of Chinese PLA General Hospital Beijing People's Republic of China; ^2^ The Fourth Unit of Third Branch Fangcang Shelter Hospital of National Exhibition and Convention Center Shanghai People's Republic of China; ^3^ State Key Laboratory of Holistic Integrative Management of Gastrointestinal Cancers and National clinical Research Center for Digestive Diseases, Xijing Hospital of Digestive Diseases Fourth Military Medical University Xi'an People's Republic of China; ^4^ Department of Nutrition, Xijing Hospital Fourth Military Medical University Xi'an People's Republic of China; ^5^ Department of Infectious Diseases, Xijing Hospital Fourth Military Medical University Xi'an People's Republic of China

**Keywords:** asymptomatic, mild symptomatic, negative conversion duration, risk factors, SARS‐CoV‐2 omicron BA.2.2 variant

## Abstract

**Objective:**

The objective of this study is to evaluate and compare clinical and virological characteristics of asymptomatic and mild symptomatic patients of severe acute respiratory syndrome coronavirus 2 (SARS‐CoV‐2) Omicron BA.2.2 variant infection and identify risk factors associated with the prolonged viral negative conversion duration.

**Methods:**

We conducted a retrospective observational study in a Shanghai (China) Fangcang shelter hospital from April 9 to May 17, 2022. The patient‐related demographic or clinical data were retrospectively recorded. Comparisons of demographic and clinical characteristics between asymptomatic and mild‐symptomatic patients were performed. Cox regression was performed to identify the risk factors of prolonged viral negative conversion duration.

**Results:**

A total of 551 patients confirmed with SARS‐CoV‐2 Omicron variant infection were enrolled in the study. Of these, 297 patients (53.9%) were asymptomatic and 254 patients (46.1%) had mild symptoms. When comparing the clinical and virological characteristics between the asymptomatic and mild symptomatic groups, several clinical parameters, including age, gender, time to viral clearance from the first positive swab, chronic comorbidities, and vaccination dose did not show statistically significant differences. In mild symptomatic patients, the median viral negative conversion duration (NCD) was 7 days (interquartile range [IQR]: 5–9), which was comparable to the median of 7 days (IQR: 5–10) in asymptomatic patients (*p* = .943). Multivariate Cox analysis revealed that patients age ≥ 60 years had a significantly higher hazard ratio (HR) for prolonged viral NCD (HR: 1.313; 95% confidence interval: 1.014–1.701, *p* = .039).

**Conclusion:**

Asymptomatic and symptomatic patients with non‐severe SARS‐CoV‐2 Omicron BA.2.2 variant infection have similar clinical features and virological courses. Old age was an independent risk factor for prolonged SARS‐CoV‐2 conversion time.

## Graphical abstract

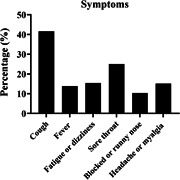

## 1 INTRODUCTION

The coronavirus disease 2019 (COVID‐19) pandemic is one of the greatest threats to human health in human history, having caused millions of deaths worldwide. In the waves of the COVID‐19 pandemic, the world is grappling with various variants of the virus, including Alpha, Beta, Gamma, Delta, and most recently, Omicron.^1^ The emergence of the Omicron (B.1.1.529) variant of severe acute respiratory syndrome coronavirus 2 (SARS‐CoV‐2) was first reported to the World Health Organization in November 2021.^2^ Due to its high transmissibility and ability to evade the immune system, Omicron has quickly replaced previous strains and becomes the dominant strain worldwide.^3^, ^4^ According to recent data summarizing US trends in variant proportions from national genomic surveillance during January 2022–May 2023 from Centers for Diseases Control and Prevention, the Omicron variants were still predominant, with >50% prevalence during the period.^5^

Over time, the Omicron variant has acquired genetic mutations, resulting in several different sublineages that have been confirmed.^6^, ^7^ Among these sublineages, the Omicron BA.2 sublineage has attracted our attention. In late February 2022, a major outbreak of the Omicron BA.2.2 variant started to spread in Shanghai, China. Although a wide range of nonpharmaceutical interventions (NPIs) were adopted in the initial stages to contain sporadic COVID‐19 outbreaks, they were ineffective in stopping this new surge.^8^ Phylogenetic analysis of SARS‐CoV‐2 viral genomes in this period revealed that all of the new viral genomes in Shanghai were predominantly clustered into the SARS‐CoV‐2 variant Omicron BA.2.2 sublineage.^9^ According to the Shanghai Municipal Health Commission data, the number of identified cases has increased to 593, 336 as of May 4, 2022.^10^ The Fangcang shelter hospitals, which can provide large numbers of hospital beds and medical care for patients who do not have severe disease, were rapidly established during this period of time.^10^ Although the epidemiological features and transmission dynamics of the Omicron BA.2.2 variant outbreak in Shanghai have been reported in previous studies, few studies have addressed the differences in clinical and virological features between asymptomatic individuals and mild symptomatic individuals infected with the Omicron BA.2.2 variant.^8^, ^9^, ^11^

The Omicron variant of SARS‐CoV‐2 has been reported to cause milder disease compared to previous predominant variants.^12^ The proportion of asymptomatic infections and the proportion of nonsevere disease among COVID‐19 Omicron patients were significantly higher than those among Delta patients.^13^ It has been proven that asymptomatic individuals play a critical role in the spread of the COVID‐19 virus.^14^, ^15^ Previous studies have shown that symptomatic cases had a longer duration of viral shedding compared with asymptomatic cases.^16^, ^17^ However, other studies have reported that there was no significant difference in the duration of viral shedding between mild symptomatic and asymptomatic infections.^18^ In addition, there is limited evidence on the viral dynamics of asymptomatic and mild symptomatic individuals with SARS‐CoV‐2 Omicron BA.2 infection, particularly for the variant BA.2.2. Thus, we conducted a retrospective observational study aimed to compare the clinical characteristics of asymptomatic and mild symptomatic cases of SARS‐CoV‐2 Omicron variant infection and identify risk factors correlated with prolonged negative conversion duration (NCD), at the Fangcang shelter hospital in Shanghai, China.

## 2 MATERIALS AND METHODS

### 2.1 Study design

Retrospective data on patients with Omicron BA.2.2 infections, who were admitted to Wards B5 and C2 of the Fangcang Hospital at the National Exhibition and Convention Center in Shanghai, China, was gathered. Inclusion criteria included the following: (1) hospitalized patients infected with the SARS‐CoV‐2 virus; (2) asymptomatic or mild symptomatic patients defined by the consensus of Guidelines for diagnosis and treatment of novelcoronavirus (2019‐nCoV) infection of China (Trial Version 9). Patients with substantial missing data were excluded from this study. This retrospective observational study was approved by the Ethics Committee of the Fangcang shelter hospital. The register number was 2022YH‐1030‐1 (May 25, 2022). All subjects provided their informed consent.

### 2.2 Setting

Before the initial surge of Omicron in 2022, Shanghai maintained a baseline level of intensive NPIs against potential outbreaks, which included stringent border control policies, symptom‐based surveillance, case isolation, tracing of close contacts (requires quarantine in separate facilities) and contacts of contacts, occupation‐based screening, targeted screening of individuals at high risk of infection, and a set of other social distancing measures such as travel restrictions and community confinement.^19^ After the Omicron variant was introduced in Shanghai, a set of additional NPIs were implemented to halt its transmission. Afterwards, the entire city entered a phased stage of lockdown on April 1. During the lockdown period, Fangcang shelter hospitals were established to temporarily accommodate the infected citizens. Once confirmed, the infected individuals were strongly recommended to transfer to nearby Fangcang shelter hospitals for basic medical observation and health monitoring.^20^ The lockdown restriction was lifted on June 1 when the daily new number of infections first declined to 10^8^.

### 2.3 Data collection and outcomes

Patients’ demographic and clinical information, such as gender, age, comorbidities, vaccine dose, diagnosis date, and admission date, were recorded on the day of admission. Pharyngeal swab specimens were acquired to detect SARS‐CoV‐2 nucleic acid daily during hospitalization. Open reading frame 1ab (ORF1ab) and nucleocapsid protein (N) were the two targeted genes of a real‐time polymerase chain reaction (RT‐PCR). The fluorescent signal counts were used to calculate cycle threshold (*C*_t_) values. Higher viral loads were a result of the decreased Ct values. When one of the *C*_t_ values of the ORF1ab and N genes was <35, RT‐PCR was considered positive. Weak positive was defined as one of the ORF1ab and N genes having a Ct value between 35 and 40. Patients would be discharged from the Fangcang shelter hospital based on the latest coronavirus pneumonia diagnosis and treatment guidelines of China: nucleic acid test negative twice in a row; improved symptoms; average body temperature for three consecutive days or more; and two consecutive negative tests 24 h apart were achieved with Ct values of ORF1ab and N genes ≥ 35. The criterion of negative conversion was two consecutive negative viral PCRs in a minimum of 24 h intervals. The negative conversion duration was defined as the interval between the initial day of the positive nasopharyngeal swab and the date of negative conversion.^21^

### 2.4 Statistical analysis

Categorical data were shown as frequencies and percentages. Continuous variables were presented as the mean and standard deviation or as the median and interquartile range (IQR). Comparisons of variables among subgroups were performed using *χ* ^2^, Fisher's exact, *t* test, and Mann–Whitney *U* test when necessary. Kaplan–Meier curves were plotted to describe the cumulative probability of negative viral conversion and comparisons stratified by factors were performed by log‐rank test. A multivariate Cox regression model was then performed with the significant factors identified by univariate analysis (*p* < .1), to determine the independent predictors of prolonged RNA‐negative conversion. Statistical analyses were performed using SPSS V.25.0. A difference with *p* < .05 was considered significant.

## 3 RESULTS

### 3.1 Baseline demographic and clinical characteristics of patients

**Figure 1:**
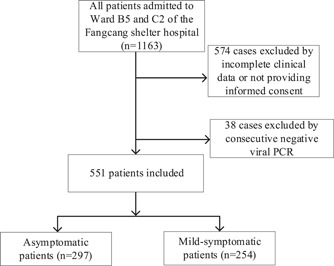
The flowchart of inclusion and exclusion criteria. PCR, polymerase chain reaction.

A total of 551 confirmed infected patients were enrolled for study in Ward B5 and C2 of the temporal hospital at the National Exhibition and Convention Center from April 9 to May 17, 2022 (Figure 1). Among those patients, 297 (53.9%) had no symptoms (asymptomatic), whereas 254 (46.1%) had only minor symptoms (mild symptomatic). Four hundred and thirty‐one patients (78.2%) were male.

**Figure 2:**
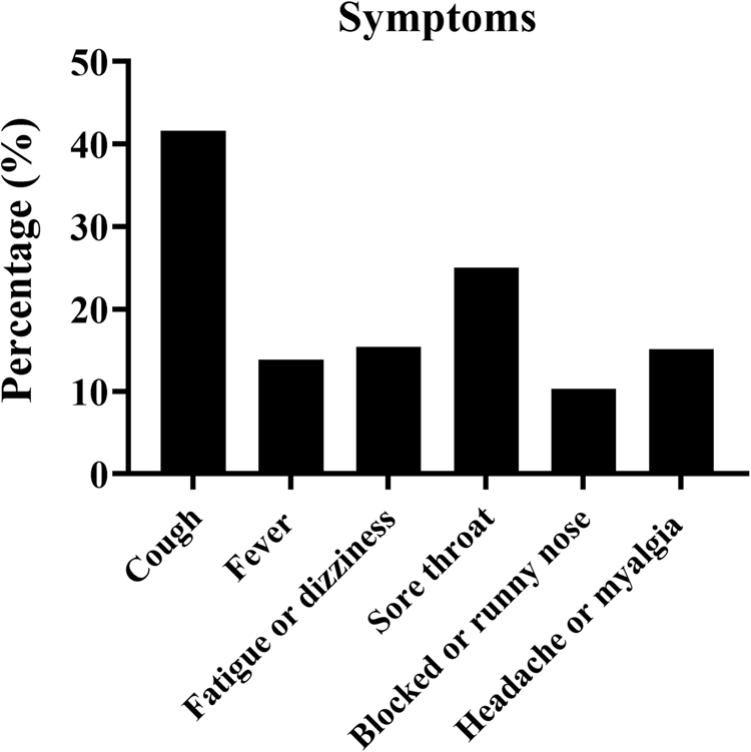
Symptoms of mild symptomatic patients with severe acute respiratory syndrome coronavirus 2 Omicron BA.2.2 variant infection in a Shanghai Fangcang shelter hospital. The most common symptom was coughing. Other common symptoms included sore throat, fatigue or dizziness, headache or myalgia, fever, and blocked or runny nose.

The mean age of patients included was 42.7 ± 15.4 years. Seventy‐four (13.4%) patients were 60 years or older. Sixty‐four patients (11.6%) had comorbidity (Table 1). Cough was the most prevalent symptom among those with mild symptoms (41.6%), followed by sore throat (25.0%), fatigue or dizziness (15.4%), headache or myalgia (15.1%), fever (13.8%), and blocked or runny nose (10.3%) (Figure 2). There were 122 (22.1%), 198 (35.9%), and 231 (42.0%) patients who had received vaccine doses of 0, 1–2, and booster, respectively (Table 1).

**Table 1:** Comparisons of demographic and clinical characteristics of asymptomatic and mild symptomatic patients with confirmed SARS‐CoV‐2 Omicron variant infection.

Variables	Overall (*n* = 551)	Asymptomatic (*n* = 297)	Mild symptomatic (*n* = 254)	*p*
Age (years), Mean ± SD	42.7 ± 15.4	42.1 ± 15.1	43.5 ± 15.7	.463
Age, *n* (%)				
<18	27 (4.9)	15 (5.1)	12 (4.7)	.156
18–60	450 (81.7)	249 (55.3%)	201 (44.7)	
≥60	74 (13.4)	33 (11.1)	41 (16.1)	
Gender, *n* (%)				
Males	431 (78.2)	236 (79.5)	195 (76.8)	.47
Females	120 (21.8)	61 (20.5)	59 (23.2)	
Time to viral clearance from first positive swab (days)				
≥7	326 (59.2)	176 (59.3)	150 (59.1)	1.000
<7	225 (40.8)	121 (40.7)	104 (40.9)	
Comorbidity				
No	487 (88.4)	269 (90.6)	218 (85.8)	.109
Yes	64 (11.6)	28 (9.4)	36 (14.2)	
Vaccinations				
0	122 (22.1)	69 (23.2)	53 (20.9)	.798
1 and 2	198 (35.9)	99 (33.3)	99 (39.0)	
3	231 (42.0)	129 (43.5)	102 (40.1)	

Abbreviation: SARS‐CoV‐2, severe acute respiratory syndrome coronavirus 2.

### 3.2 Comparison between mild symptomatic patients versus asymptomatic patients

We compared baseline demographic and clinical characteristics between asymptomatic and mild symptomatic patients. As shown in Table 1, the mean age of asymptomatic patients was 42.1 ± 15.1 and the mean age of mild‐symptomatic patients was 43.5 ± 15.7. A higher proportion of patients aged ≥ 60 years was observed in mild symptomatic patients than in asymptomatic patients (16.1% vs. 11.1%). The age distribution of the two groups did not, however, differ significantly (*p* = .156). In terms of percentage, more symptomatic cases (14.2% vs. 9.4%, *p* = .109) than asymptomatic cases (9.4%) had at least one chronic comorbidity. Besides, there was no statistically significant difference in gender distribution, the median viral shedding time, and vaccination doses between the two groups (all *p* > .05). We also compared the *C*_t_ values of ORF1ab and N genes on the admission day (Supporting Information: Figure 1). In the asymptomatic group, the mean *C*_t_ values of ORF1ab were 33.2 ± 5.0, which was noticeably higher than the mildly symptomatic group's mean *C*_t_ values of 32.2 ± 5.0 (*p* = .023). Regarding the *C*_t_ values of the N gene, a significant difference was also discovered (*p* = .044).

### 3.3 Factors associated with prolonged RNA‐negative conversion duration

**Figure 3:**
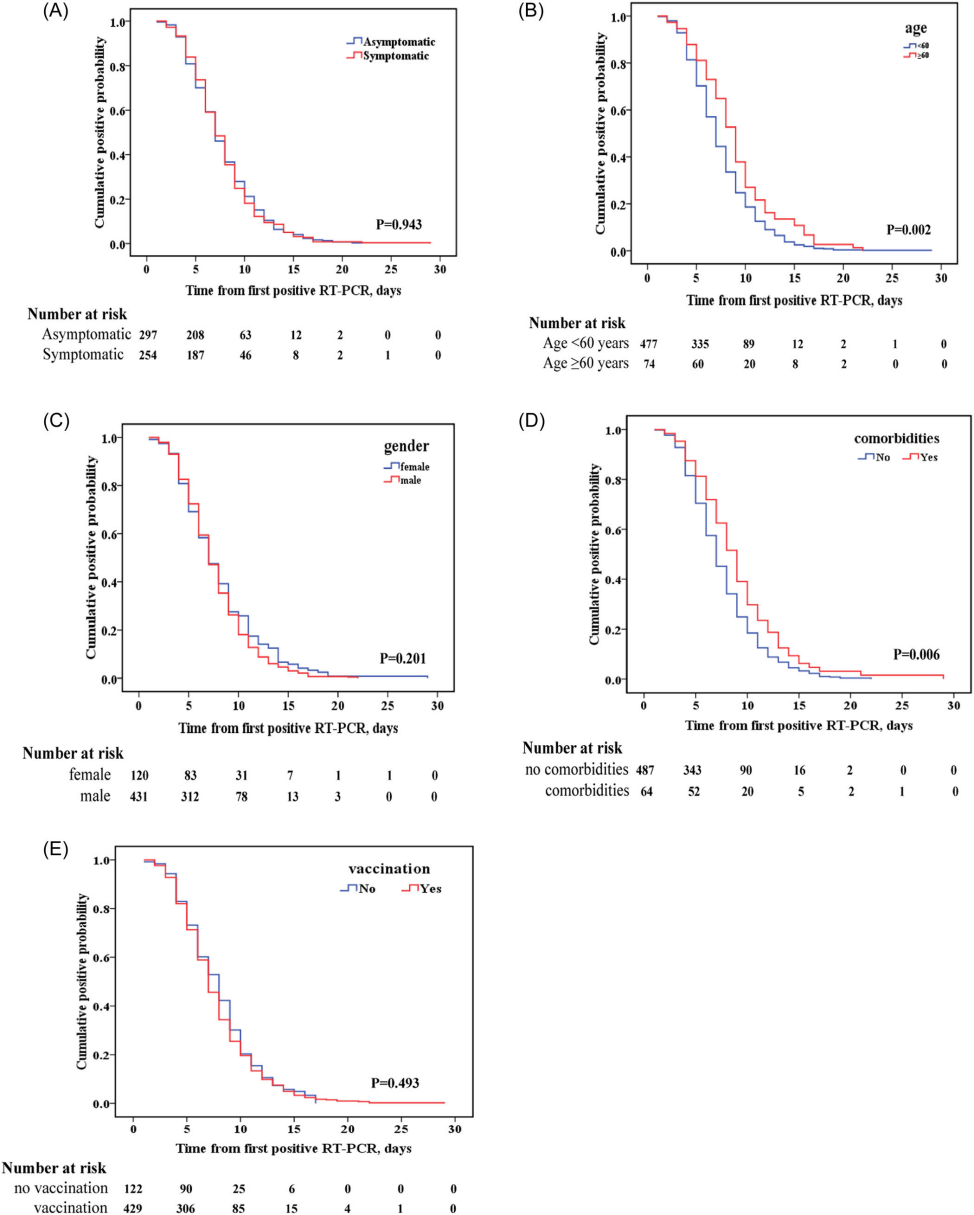
Kaplan–Meier curves for negative conversion time of 551 patients with severe acute respiratory syndrome coronavirus 2 Omicron BA.2.2 variant infection based on symptom (A), age (B), gender (C), comorbidities (D) and vaccination (E). RT‐PCR, real‐time polymerase chain reaction.

The median conversion time from initial positive RT‐PCR to SARS‐Cov‐2 negative status (negative conversion duration, NCD) was 7 days in all patients (IQR: 5–10 days). Kaplan–Meier curves showed the cumulative probability of negative viral conversion stratified by different subgroups. As shown in Figure 3A, the median NCD in asymptomatic patients was 7 days (IQR: 5–10), which was comparable to the median NCD in mild‐symptomatic patients of 7 days (IQR: 5–9) (*p* = .943). Patients aged ≥ 60 years had a median time to RNA viral conversion of 9 days (IQR: 6–11 days), which was substantially longer than patients aged < 60 years, who had a median time of 7 days (IQR: 5–9) (Figure 3B). Additionally, patients with chronic comorbidities had a significantly longer median negative conversion time than patients without chronic comorbidities (Figure 3D). Individuals with chronic comorbidities experienced a median duration of 9 days (IQR: 6–11 days), whereas individuals without such conditions experienced a median duration of 7 days (IQR: 5–9 days). There was no significant difference in the duration of SARS‐CoV‐2 RNA‐negative conversion according to gender (Figure 3C) and vaccination status (Figure 3E).

As shown in Table 2, the univariate Cox regression analysis revealed that patients with an age ≥ 60 years (hazard ratio [HR]: 1.56; 95% confidence interval [95% CI]: 1.23–1.97); *p* = .0002) and chronic comorbidities (HR: 1.38; 95% CI: 1.15–1.83; *p* = .01) had prolonged negative conversion duration compared with those patients without the above factors. In multivariate Cox regression, only age ≥ 60 years (HR: 1.31; 95% CI: 1.01–1.70); *p* = .039) was identified as an independent risk factor for prolonged negative conversion duration of viral RNA in asymptomatic and mild symptomatic patients with Omicron BA.2.2 variant infection.

**Table 2:** Factors associated with prolonged negative conversion of viral RNA by Cox regression analysis.

Variables	Univariate Cox regression analysis		Multivariate Cox regression analysis	
	HR (95% CI)	*p*	HR (95% CI)	*p*
Asymptomatic versus symptomatic	1.006 (0.850–1.189)	.949		
Age (≥60 vs. <60)	1.559 (1.231–1.974)	.002	1.313 (1.014–1.701)	.039
Gender (males vs. females)	1.126 (0.918–1.381)	.254		
Comorbidities (yes vs. no)	1.378 (1.146–1.826)	.006	1.264 (0.957–1.669)	.099
Vaccination (yes vs. no)	1.065 (0.871–1.302)	.539		

Abbreviations: CI, confidence interval; HR, hazard ratio.

## 4 DISCUSSION

The study enrolled a total of 551 cases infected with the SARS‐CoV‐2 Omicron BA.2.2 variant. Of these, 297 patients (53.9%) were asymptomatic and 254 patients (46.1%) were mild symptomatic. The results show that nonsevere patients infected with the Omicron BA.2.2 variant had a high proportion of asymptomatic infections. Compared to the proportion of asymptomatic infection in nonsevere COVID‐19 cases in 2020 (29.4%), there were more asymptomatic infections during the omicron BA.2.2 variant wave.^22^ If asymptomatic patients are not identified and quarantined in time, they could become moving sources of further infection and lead to massive transmission of disease. The high prevalence of asymptomatic infection is probably a major contributing factor to the widespread, rapid dissemination of this variant globally.^23^

Notably, among 254 symptomatic patients, 76.1% presented at least two symptoms. The most common symptom was cough (41.6%), followed by sore throat (25.0%). This observation is consistent with previous studies, which found that upper respiratory tract symptoms (cough, sore throat) were more common in patients with Omicron infection than those with delta variant infection.^24^, ^25^ Our results found that 225 (40.8%) patients achieved a virus‐free status within 7 days (data not shown). In contrast, early viral variants required a longer time to be cleared from the upper respiratory tract.^26^, ^27^, ^28^ In this study, the median duration of negative conversion duration was 7 days for both asymptomatic and mild symptomatic patients, which might indicate that asymptomatic and mild symptomatic patients carry the virus and are infectious for a similar duration of time. It was also worth noting that the symptomatic patients had significantly lower *C*_t_ values than the asymptomatic patients in the study. Our finding of lower *C*_t_ values in symptomatic patients and longer duration of viral shedding in older patients is in line with observations of higher household transmission rates from symptomatic versus asymptomatic index cases and index patients older than 60 years compared with adults, adolescents, and children under the age of 12 years.^29^

Based on our evaluation, the vaccination coverage rate was 77.9% for all included patients. However, the rate of booster doses was relatively low, at 42.0%. Reports from China also indicated that about the half of the population have received three doses of the vaccine_._ ^30^ The low booster dose coverage might be one possible reason why the vaccination protection effect against the Omicron variant was less efficient.^31^ Another reason might be that the usage of inactivated vaccines provided less sufficient protection, especially against Omicron variant infection.^32^ Nonetheless, other studies demonstrated that inactivated vaccines could still provide protection against severe outcomes.^33^, ^34^

It was also worth noting that both the baseline demographics and the clinical characteristics were not significantly different between asymptomatic and mild symptomatic individuals. Further, Cox regression analysis confirmed that the symptomatic status was not a risk factor for prolonged duration of SARS‐CoV‐2 RNA‐negative conversion. Thus, our results suggested that symptomatic status was not associated with major clinical characteristics like the duration of viral shedding in non‐severe patients of Omicron BA.2.2 infection. We speculate that the above conclusion could be partially explained by the indistinguishable difference in viral dynamic between mild symptomatic and asymptomatic patients, which was also reported in previous study.^35^

In our study, univariate analysis results showed that chronic comorbidities were a risk factor for prolonged viral shedding duration, but no significant difference was found in the multivariate Cox analysis. In addition, it was demonstrated that old age is an independent risk factor associated with prolonged viral shedding duration, which was in line with previous studies.^36^, ^37^ However, a retrospective study on a large sample of Fangcang hospital cases infected by the SARS‐CoV‐2 omicron variant showed that age, fever, cough, fatigue, and comorbidity are predictors for the deterioration of SARS‐CoV‐2 omicron variant infection.^38^ In that study, different from ours, the researchers compared the clinical characteristics between non‐severe patients and severe patients, and identified the risk factors for severe COVID‐19 infection. Generally speaking, elderly people are more vulnerable to be infected with SARS‐CoV‐2 virus due to their weaker immune systems. Therefore, we speculated that age‐related comorbidities appear to be a synergistic factor rather than an independent risk factor for prolonged viral shedding. These results also indicate that elderly patients require additional monitoring and medical attention during the COVID‐19 pandemic.

In the context of the vaccination strategy adopted until March 2022, it was estimated that the introduction of the Omicron variant would overwhelm the healthcare system with an estimated burden of 15.6 times the available intensive care unit capacity.^30^ If no strict public health measures were taken, such as large‐scale viral nucleic acid and antigen screening, quarantine of infected cases, and close contacts in shelter hospitals such as Fangcang shelter hospitals and hotels, respectively, and lockdown of districts with severe outbreak, the number of severe to critical cases and the resultant death toll could be high among the older people without vaccination.^8^ Therefore, the strict and comprehensive containing strategies in Shanghai were actually implemented to reduce the number of people infected, and to provide early diagnosis and appropriate treatment for severe COVID‐19 patients. The ultimate goal was to reduce the case fatality rate and buy time for full vaccination coverage, especially for older people.

Our study had some limitations. First, the variables included in our study are limited due to its retrospective nature. Some variables, such as body mass index, laboratory tests, imaging examinations, and some risk factors associated with respiratory tract infections were not included in the database. Therefore, our findings need to be confirmed in cohorts that have more detailed variables included. Second, the study only compared the characteristics of asymptomatic and mild symptomatic COVID‐19 patients. Previous study showed that significant differences were identified between mild and more severe patients.^39^ Therefore, further research, enrolling patients with varying degrees of COVID severity and group of uninfected control, may be necessary. Third, the test for COVID‐19 in this study was based on RT‐PCR, which measures the corresponding Ct values. However, RT‐PCR may produce both false‐negative and false‐positive results. Therefore combining the results of RT‐PCR with the results of serologic tests or viral culture can provide more reliable information. Fourth, The study is not gender balanced, with 120 women and 431 men. To maintain favorable orders in Fangcang shelter hospital, the patients admitted into Fangcang shelter hospital were mainly distributed according to the gender. In our study, all the patients were from Ward B5 and C2 of the Fangcang shelter hospital. Ward B5 was the larger‐sized zone, where male‐patients were admitted in particular. In contrast, Ward C2 was the smaller‐sized zone, where female‐patients were admitted in particular. Therefore, inevitably this may bring the problem of gender bias.

In conclusion, asymptomatic and symptomatic patients with nonsevere SARS‐CoV‐2 Omicron BA.2.2 variant infection have similar clinical features and virological courses. Symptomatic status was not associated with the clinical characteristics such as the duration of viral shedding in asymptomatic and mild symptomatic patients of Omicron BA.2.2 infection. Old age was identified as an independent risk factor for prolonged SARS‐CoV‐2 conversion time. A better understanding of the clinical and virological features of the Omicron BA.2.2 subvariant infected individuals using our data could provide valuable insights for dealing with the ongoing Omicron‐predominant COVID‐19 pandemic.

## AUTHOR CONTRIBUTIONS

*Conception and design*: Zhangqian Chen. *Acquisition of data*: Zhangqian Chen, Liangliang Wang, and Xiaoyu Kang. *Analyses and interpretation of data*: Lin Zhang, Xiaoyu Kang, Liangliang Wang, and Rui Yan. *Critical revision of the manuscript for important intellectual content*: Xiaoyu Kang and Yanglin Pan. *Administrative and material support*: Jiuping Wang. *Final approval of the version submitted*: all authors. All authors read and approved the final manuscript.

## CONFLICT OF INTEREST STATEMENT

The authors declare no conflict of interest.

## Supporting information

Supporting Information.

Supporting Information.

Supporting Information.

## Data Availability

The data that support the findings of this study are available from the corresponding author upon reasonable request.
