# Supplementary material for: Clinical and virological features of asymptomatic and mild symptomatic patients with SARS‐CoV‐2 Omicron infection at Shanghai Fangcang shelter hospital
Source: Immun Inflamm Dis. 2023 Sep 27;11(9):e1033. doi: 10.1002/iid3.1033 (PMC10524057; doi:10.1002/iid3.1033)
Supplement: Supplementary file 1 — Supporting Information. [file IID3-11-e1033-s002.docx]

Supplementary table 1. Comparisons of parameters based on Ct values on hospitalization day.

| Variables | Ct value <30 (n=244) | Ct value≥ 30 and <35 (n=209) | P value |
| --- | --- | --- | --- |
| Gender, n (%) |  |  | 0.536 |
| Female | 50 (20.5%) | 38(18.2%) |  |
| Male | 194 (79.5%) | 171(81.8%) |  |
| Age, mean (SD) | 44.4±16.7 | 42.5±15.4 | 0.178 |
| Comorbidity |  |  | 0.238 |
| No | 222(91.0%) | 183 (87.6%) |  |
| Yes | 22(9.0%) | 26 (12.4%) |  |
| Severity of COVID-19 |  |  | 0.198 |
| Asymptomatic | 123 (51.0%) | 118 (56.5%) |  |
| Mild-symptomatic | 121(49.0%) | 91 (43.5%) |  |
| NCD, mean (SD) | 7(5-10) | 7 (5-9) | 0.015 |
