# Supplementary figures and images for: Clinical and virological features of asymptomatic and mild symptomatic patients with SARS‐CoV‐2 Omicron infection at Shanghai Fangcang shelter hospital
Source: Immun Inflamm Dis. 2023 Sep 27;11(9):e1033. doi: 10.1002/iid3.1033 (PMC10524057; doi:10.1002/iid3.1033)

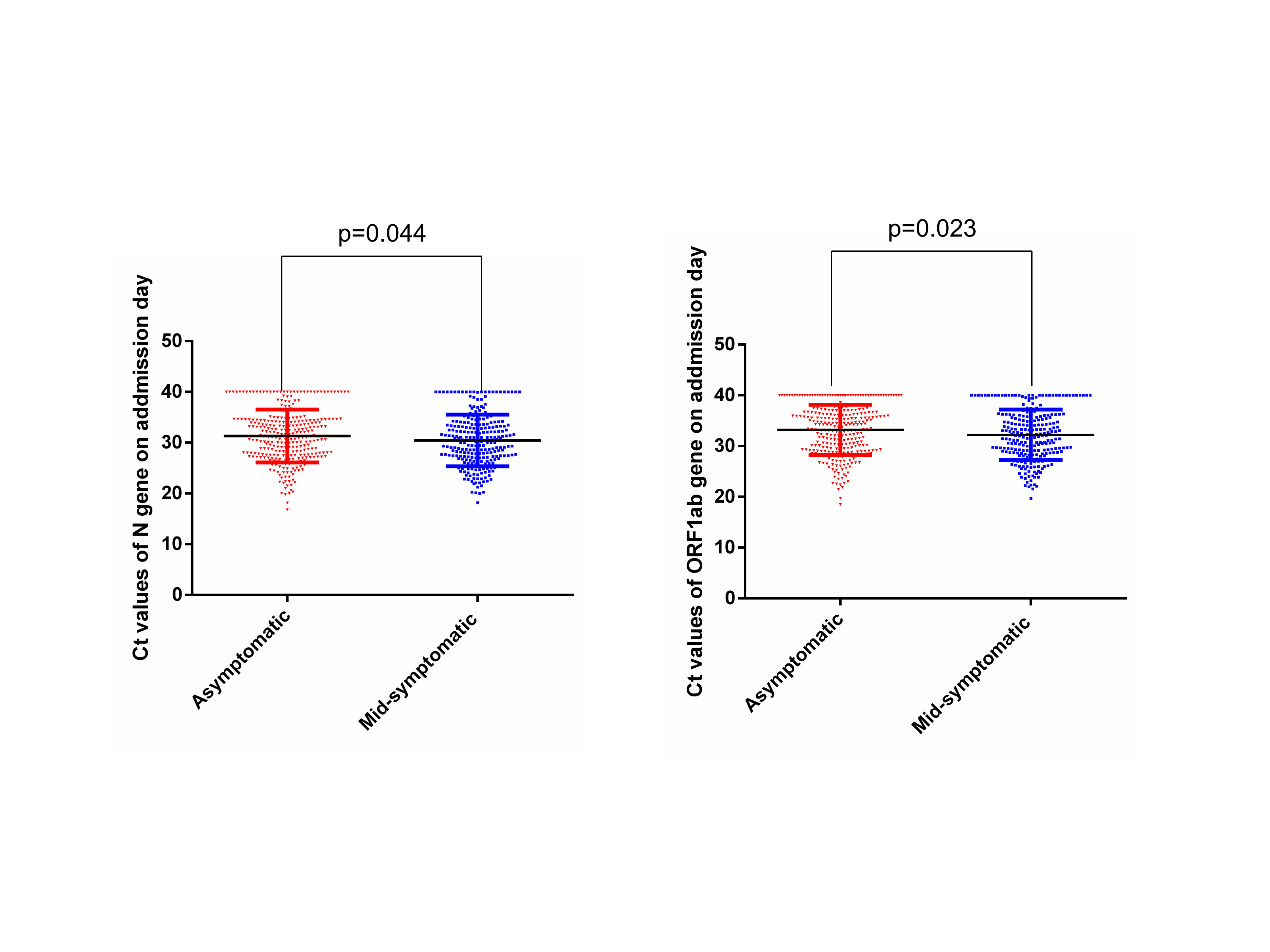

Supplement: Supplementary file 2 — Supporting Information. [file IID3-11-e1033-s003.tif]
