# Supplementary material for: Clinical and virological features of asymptomatic and mild symptomatic patients with SARS‐CoV‐2 Omicron infection at Shanghai Fangcang shelter hospital
Source: Immun Inflamm Dis. 2023 Sep 27;11(9):e1033. doi: 10.1002/iid3.1033 (PMC10524057; doi:10.1002/iid3.1033)
Supplement: Supplementary file 3 — Supporting Information. [file IID3-11-e1033-s001.docx]

**Figure legend:**

supplementary figure 1: The Cycle threshold (CT) values of N genes (left) and ORF1ab (right) on the admission day.
